# Cardiovascular autonomic dysfunction in Parkinson's disease: associations with disease severity and the modulatory role of dopaminergic therapy

**DOI:** 10.1055/s-0046-1825525

**Published:** 2026-07-14

**Authors:** Yogesh Kumar, Gunjan Kumar, Nirangjhana Sivasubramanian

**Affiliations:** 1 All India Institute of Medical Sciences Patna, Department of Physiology, Patna BR, India.; 2 Patna Medical College and Hospital, Department of Neurology, Patna BR, India.; 3 All India Institute of Medical Sciences Patna, Department of Physiology, Doctorate of Medicine Program in Clinical and Interventional Physiology, Patna BR, India.

**Keywords:** Parkinson Disease, Autonomic Nervous System Diseases, Heart Rate, Hypotension, Orthostatic, Dopamine Agents, Baroreflex

## Abstract

**Background:**

Parkinson's disease (PD) is a complex neurodegenerative disorder characterized by both motor and non-motor symptoms, with autonomic dysfunction significantly impacting patients' quality of life. Despite its recognized importance, comprehensive evaluation of autonomic dysfunction in PD remains limited.

**Objective:**

To assess cardiovascular autonomic dysfunction in PD, explore its association with disease severity, and examine the modulatory effects of dopaminergic therapy.

**Methods:**

A cross-sectional study was conducted involving 60 PD patients and 30 age- and sex-matched healthy controls. Autonomic function was evaluated using standardized cardiovascular tests, including deep breathing, the Valsalva maneuver, and head-up tilt. Heart rate variability, baroreflex sensitivity, and orthostatic blood pressure responses were measured. Parkinson's disease patients were evaluated in both dopaminergic medication ON and OFF states. Correlations between autonomic parameters and disease severity were analyzed.

**Results:**

Parkinson's disease patients demonstrated significant autonomic impairment compared to controls ( *p* < 0.001). Standard deviation of NN intervals (SDNN) (21.3 ± 8.7 vs. 32.5 ± 10.2 ms), baroreflex sensitivity (4.2 ± 1.8 vs. 7.1 ± 2.3 ms/mmHg), and heart rate response to deep breathing (8.3 ± 3.2 vs. 13.7 ± 4.1 bpm) were reduced, while systolic blood pressure drop on tilt increased (22.5 ± 12.8 vs. 8.3 ± 5.7 mmHg). Autonomic impairment correlated with disease severity. Dopaminergic medication showed modest but significant improvements in heart rate variability ( *p* = 0.032), baroreflex sensitivity ( *p* = 0.041), and orthostatic response ( *p* = 0.025).

**Conclusion:**

Parkinson's disease patients exhibit marked cardiovascular autonomic dysfunction correlating with disease severity. The modest modulatory effects of dopaminergic medication on autonomic parameters suggest complex dopaminergic-autonomic interactions and supports autonomic parameters as potential markers of disease progression.

## INTRODUCTION

Parkinson's disease (PD) is a progressive neurodegenerative disorder affecting approximately 1 to 2% of individuals over 65 years of age, making it the second most common neurodegenerative disease worldwide. 1 While primarily recognized for motor symptoms including tremor, rigidity, bradykinesia, and postural instability, non-motor manifestations profoundly impact patients' quality of life. 2

Among the non-motor symptoms, autonomic dysfunction represents a particularly debilitating feature of PD, affecting 80 to 90% of patients during the course of the disease. 3 It encompasses diverse symptoms affecting multiple organ systems: cardiovascular dysautonomia (orthostatic hypotension, supine hypertension), gastrointestinal disturbances (constipation, dysphagia), urogenital dysfunction (urinary urgency, nocturia, sexual dysfunction), thermoregulatory abnormalities (hyperhidrosis, heat intolerance), and pupillomotor changes. 4

The autonomic nervous system comprises sympathetic and parasympathetic divisions regulating involuntary functions through complex neural networks. 5 In PD, dysfunction results from widespread deposition of Lewy bodies and Lewy neurites containing aggregated alpha-synuclein in central autonomic nuclei and peripheral autonomic structures. 6

According to the Braak staging hypothesis, alpha-synuclein pathology may begin in peripheral autonomic structures, including the cardiac sympathetic nerves and enteric nervous system, before spreading centrally, 7 explaining why autonomic symptoms like constipation often precede motor symptoms. 8 Cardiovascular autonomic dysfunction reflects impairment across multiple levels: parasympathetic dysfunction manifests as reduced heart rate variability and abnormal cardiovascular reflexes, while sympathetic dysfunction causes orthostatic hypotension. 9 Cardiac sympathetic denervation represents a prominent autonomic feature in PD. 10

Orthostatic hypotension affects 30 to 60% of PD patients, increasing risk of falls, syncope, cognitive impairment, and mortality. 11 Autonomic symptoms often respond poorly to dopaminergic therapies and may be exacerbated by certain medications. 12 Despite high prevalence, systematic autonomic assessment is not routine in clinical practice. 13 The relationship between autonomic dysfunction and disease severity remains incompletely characterized, with inconsistent results regarding dopaminergic therapy effects. 14

While several studies have examined autonomic dysfunction in PD, significant knowledge gaps remain. Previous studies have primarily focused on either isolated autonomic parameters or small patient cohorts. Alonso et al. 15 examined heart rate variability in relation to PD risk but did not assess the effects of medication states on autonomic parameters in established PD patients. Kallio et al. 16 investigated nocturnal cardiac autonomic regulation in 18 PD patients but did not examine daytime cardiovascular reflexes or medication effects. Velseboer et al. 11 investigated orthostatic hypotension prevalence without comprehensive cardiovascular reflex testing across multiple autonomic domains. Furthermore, the relationship between autonomic dysfunction and disease severity has yielded inconsistent results across studies, 13 17 18 partly due to heterogeneous methodologies and lack of comprehensive autonomic assessment protocols. Critically, the modulatory effects of dopaminergic therapy on autonomic parameters remain controversial. While Goldstein et al. 19 demonstrated cardiac sympathetic denervation in PD and Palma and Kaufmann 14 reviewed treatment approaches for autonomic dysfunction, systematic studies comparing ON and OFF medication states with comprehensive autonomic testing remain limited. These inconsistencies may reflect differences in medication states during testing, disease duration, disease severity, and autonomic testing protocols employed.

Based on the pathophysiological understanding of PD and previous limited evidence, we hypothesized that PD patients would demonstrate significant impairment across multiple cardiovascular autonomic parameters compared to age-matched controls. Furthermore, the degree of autonomic dysfunction might correlate positively with disease severity markers (part III of the Unified Parkinson's Disease Rating Scale UPDRS III score, Hoehn and Yahr [H&Y] stage, and disease duration), and dopaminergic medication would show measurable modulatory effects on autonomic parameters, with improvement in the ON compared to OFF state. Hence the present study was conducted to address these hypotheses and existing knowledge gaps by:

- providing comprehensive assessment of cardiovascular autonomic function using standardized, validated protocols in a well-characterized PD cohort (n = 60);
- systematically determining whether autonomic dysfunction correlates with multiple disease severity markers using robust statistical methods including partial correlation analyses; and
- examining dopaminergic medication effects by comparing ON and OFF states in the same patients, thereby controlling for inter-individual variability.

These objectives aim to enhance understanding of autonomic dysfunction in PD and inform clinical management strategies.

## METHODS

### Study design and setting

The present cross-sectional observational study was conducted at the Autonomic Function Laboratory, Department of Physiology, in collaboration with the Department of Neurology, AIIMS Patna, between January 2022 and December 2023.

### Sample size determination

Sample size calculation was based on expected heart rate variability differences between PD patients and controls using the formula n = (Zα/2 + Zβ) ^2^ × 2σ ^2^ /(μ1 − μ2) ^2^ . With Zα/2 = 1.96, Zβ = 0.84, σ = 12 ms, and expected mean difference = 10 ms, the minimum required sample size was 23 per group. 20 We enrolled 60 PD patients and 30 controls for adequate power and accounting for potential issues, consistent with published studies. 21

### Participant selection

The study included 60 patients with idiopathic PD recruited from the Department of Neurology and 30 age- and sex-matched healthy volunteers. Parkinson's disease patients aged 40 to 75 years diagnosed according to the Movement Disorder Society criteria 22 with disease duration ≥ 1 year, on stable dopaminergic regimen ≥ 6 months, and able to provide informed consent were included. Controls aged 40 to 75 years without neurological/psychiatric disorders, significant cardiovascular disease, or on medications affecting autonomic function were recruited.

Exclusion criteria (both groups) included secondary or atypical parkinsonism, significant autonomic dysfunction from causes other than PD (diabetes mellitus with neuropathy, primary autonomic failure, multiple system atrophy), cardiac arrhythmias, pacemakers, or significant structural heart disease, uncontrolled hyper or hypotension, chronic kidney disease or hepatic dysfunction, current or recent (< 6 months) cerebrovascular events, severe cognitive impairment (Mini-Mental State Examination [MMSE] score < 24). 23

Use of medications affecting cardiovascular autonomic function – including anticholinergic medications (e.g., oxybutynin, tolterodine, trihexyphenidyl), alpha-adrenergic blockers (e.g., tamsulosin, doxazosin, terazosin for benign prostatic hyperplasia), beta-blockers and other antihypertensive agents directly affecting heart rate variability, tricyclic antidepressants (e.g., amitriptyline, nortriptyline, imipramine), and antiarrhythmic medications – constituted additional exclusion criteria for both groups.

Patients on the following medications were included in the study if doses had been stable for ≥ 3 months: dopaminergic therapies (levodopa/carbidopa), dopamine agonists (pramipexole, ropinirole), monoamine oxidase-B (MAO-B) inhibitors (rasagiline, selegiline), catechol-O-methyltransferase (COMT) inhibitors (entacapone), selective serotonin reuptake inhibitors (SSRIs) or serotonin-norepinephrine reuptake inhibitors (SNRIs) for depression, as these have minimal direct cardiovascular autonomic effects at therapeutic doses.

### Clinical assessment

All participants underwent comprehensive evaluation including detailed medical history, physical examination, neurological assessment, and MMSE. 23 Parkinson's disease patients received disease-specific assessments: UPDRS part III, 24 Modified H&Y stage, 25 Schwab and England Activities of Daily Living Scale (SE-ADL), 26 medication documentation with levodopa equivalent daily dose calculation, 27 and disease duration recording.

### Autonomic function testing

All tests were performed in a temperature-controlled laboratory (22–24°C) with dimmed lighting. Participants avoided caffeine, alcohol, and heavy meals for ≥ 3 hours and strenuous exercise for 24 hours before morning testing (9 AM–12 PM). After 15 minutes of supine rest, continuous electrocardiogram (ECG) and blood pressure monitoring using the PowerLab system (AD Instruments) was initiated.

### Deep breathing test

Six cycles at 6 breaths/minute (5-second inspiration, 5-second expiration) were performed in supine position under visual guidance. 28 Heart rate response (maximum–minimum per cycle averaged) and expiration:inspiration ratio were measured.

### Valsalva maneuver

Three trials of forced expiration at 40 mmHg for 15 seconds with 3-minute rest intervals were performed. 29 Valsalva ratio (maximum heart rate phase II/minimum heart rate phase IV) and blood pressure responses were recorded.

### Head-up tilt test

Participants were tilted to 70 degrees. 30 Heart rate and blood pressure were monitored for 5 minutes supine (baseline) and 5 minutes tilted. Changes in systolic/diastolic blood pressure and heart rate were measured. Orthostatic hypotension was defined as ≥ 20 mmHg systolic or ≥ 10 mmHg diastolic decrease within 3 minutes. 31

### Heart rate variability analysis

From 5-minute supine ECG recordings, 32 time-domain parameters, standard deviation of NN intervals (SDNN), and root mean square of successive differences (RMSSD) were calculated, and frequency-domain parameters (low frequency (LF), high frequency (HF) power, LF/HF ratio) were analyzed.

### Baroreflex sensitivity

Baroreflex sensitivity was calculated using the sequence method from continuous blood pressure and heart rate recordings during supine rest, 33 identifying ≥ 3 consecutive beats in which progressive increase in systolic blood pressure was followed by R-R interval lengthening, expressed as ms/mmHg or vice versa.

### Medication state testing

Parkinson's disease patients underwent autonomic function testing in two medication states: OFF and ON states. In OFF state, testing was performed after overnight withdrawal of dopaminergic medications: ≥ 12 hours for regular-release levodopa formulations, ≥ 24 hours for controlled-release levodopa formulations, ≥ 48 hours for long-acting dopamine agonists. Monoamine oxidase-B and COMT inhibitors were continued due to their long half-lives.

In ON state, testing was performed 60 to 90 minutes after the patient's usual morning dose of dopaminergic medications (when patients typically experience optimal motor response). Testing was performed in OFF state first, followed by ON state, with a minimum 2-hour interval between testing sessions. The order was not randomized due to practical and ethical considerations (patients preferred OFF state testing early to minimize discomfort).

### Ethical considerations

The study received Institutional Ethics Committee approval (AIIMS/Pat/IEC/2021/789) and followed the principles of the Declaration of Helsinki. 34 Written informed consent was obtained from all participants (and legally authorized representatives for cognitively impaired patients). Tests were performed under medical supervision with emergency equipment available and terminated if symptomatic hypotension occurred.

### Statistical analysis

Data analysis was performed using the IBM SPSS Statistics for Windows (IBM Corp.), version 26.0 software. The Shapiro-Wilk test was used to assess normality of continuous variables. Continuous variables are presented as mean ± standard deviation (SD) and categorical variables as frequencies and percentages. Between-group comparisons such as differences in demographic characteristics and autonomic parameters between PD patients and healthy controls were analyzed using independent samples t-tests for continuous variables and Chi-squared tests (or Fisher's exact test when expected cell counts < 5) for categorical variables.

Within-subject comparisons such as differences in autonomic parameters between medications OFF and ON states in PD patients were analyzed using paired t-tests. Correlation analyses were performed to examine relationships between autonomic parameters and multiple variables including disease severity markers (UPDRS III score, H&Y stage), disease duration, levodopa equivalent daily dose (LEDD), and demographic variables (age, sex, Body Mass Index). Pearson's correlation coefficients were used for normally distributed continuous variables, and Spearman's rank correlation for non-normally distributed variables or ordinal data. Partial correlation analyses were conducted to adjust for potential confounders including age, disease duration, and LEDD where applicable. All statistical tests were two-tailed, and *p* < 0.05 was considered statistically significant. Post-hoc power analysis confirmed > 80% statistical power for primary outcomes at α = 0.05.

## RESULTS

All 90 participants (60 PD patients, 30 controls) completed the study without adverse events. The PD and control groups were well-matched for age, sex distribution, and body mass index. Parkinson's disease patients had mean disease duration 7.3 years, UPDRS III score 22.7 (mild-moderate motor severity), and H&Y stage distribution: stage 1 (13.3%), stage 2 (53.3%), stage 2.5 (20.0%), and stage 3 (13.3%), with a mean H&Y stage of 2.3. The mean Schwab and England Activities of Daily Living (SE-ADL) score was 78.5%, indicating relatively preserved functional independence. All patients were receiving dopaminergic therapy. All 60 patients (100%) were on levodopa/carbidopa preparations. Additionally, 38 patients (63.3%) were on dopamine agonists, 28 patients (46.7%) were on MAO-B inhibitors, and 15 patients (25.0%) were on COMT inhibitors. The mean levodopa equivalent daily dose (LEDD) was 545 ± 285 mg/day (range: 200–1,250 mg/day) as shown in Table 1 .

**Table 1:** Demographic and clinical characteristics of study participants

Characteristic	PD patients	Controls	*p* -value
**Demographics**			
**Age (years)**	62.5 ± 8.3	61.8 ± 7.9	0.698
**Sex (male/female)**	35/25	17/13	0.892
** Body Mass Index (kg/m ^2^ ) **	24.8 ± 3.2	25.1 ± 2.9	0.673
**Disease characteristics**			
**Disease duration (years)**	7.3 ± 4.2	**–**	**–**
**UPDRS III score**	22.7 ± 9.8	**–**	**–**
**Hoehn and Yahr Stage (n, %)**			
**Stage 1**	8 (13.3%)	**–**	**–**
**Stage 2**	32 (53.3%)	**–**	**–**
**Stage 2.5**	12 (20.0%)	**–**	**–**
**Stage 3**	8 (13.3%)	**–**	**–**
**Stage 4**	0 (0%)	**–**	**–**
**Mean H&Y stage**	2.3 ± 0.7	**–**	**–**
**SE-ADL score (%)**	78.5 ± 12.3	**–**	**–**
**Dopaminergic therapy**			
**Levodopa therapy (n, %)**	60 (100%)	**–**	**–**
**Dopamine agonist (n, %)**	38 (63.3%)	**–**	**–**
Pramipexole	25 (41.7%)	**–**	**–**
Ropinirole	13 (21.7%)	**–**	**–**
**MAO-B inhibitor (n, %)**	28 (46.7%)	**–**	**–**
**COMT inhibitor (n, %)**	15 (25.0%)	**–**	**–**
**LEDD (mg/day)**	545 ± 285	**–**	**–**
**Range**	200–1,250	**–**	**–**
**Concomitant medications**			
** Antidepressants (SSRI/SNRI) (n, %)**	18 (30.0%)	3 (10.0%)	

Abbreviations: COMT, catechol-O-methyltransferase; H&Y, Hoehn and Yahr; LEDD, levodopa equivalent daily dose; MAO-B, monoamine oxidase B; SE-ADL, Schwab and England Activities of Daily Living; SNRI, serotonin-norepinephrine reuptake inhibitor; SSRI, selective serotonin reuptake inhibitor; UPDRS, Unified Parkinson's Disease Rating Scale.

Note: Data presented as mean ± SD, count (percentage), or median (IQR).

Parkinson's disease patients demonstrated significant impairment across all autonomic parameters compared to controls ( Table 2 ). Time-domain heart rate variability showed substantial reductions: SDNN decreased 34.5% and RMSSD 35.3% compared to controls, indicating marked impairment of autonomic modulation and parasympathetic activity. Baroreflex sensitivity showed severe 40.8% reduction. Dynamic cardiovagal testing confirmed profound impairment: heart rate response to deep breathing reduced 39.4%, E:I ratio decreased 7.4%, and Valsalva ratio reduced 16.6%.

**Table 2:** Comparison of autonomic parameters between PD patients and controls

Parameter		PD patients (n = 60)	Controls (n = 30)	Difference (%)	*p* -value
**Cardiovagal function**	**SDNN (ms)**	21.3 ± 8.7	32.5 ± 10.2	-34.5	< 0.001
	**RMSSD (ms)**	18.7 ± 7.2	28.9 ± 8.5	-35.3	< 0.001
	**BRS (ms/mmHg)**	4.2 ± 1.8	7.1 ± 2.3	-40.8	< 0.001
	**HR response to deep breathing (bpm)**	8.3 ± 3.2	13.7 ± 4.1	-39.4	< 0.001
	**E:I ratio**	1.12 ± 0.08	1.21 ± 0.10	-7.4	< 0.001
	**Valsalva ratio**	1.21 ± 0.15	1.45 ± 0.18	-16.6	< 0.001
**Adrenergic function**	**SBP drop on tilt (mmHg)**	22.5 ± 12.8	8.3 ± 5.7	+171.1	< 0.001
	**DBP drop on tilt (mmHg)**	11.8 ± 7.3	4.2 ± 3.1	+181.0	< 0.001
	**HR increase on tilt (bpm)**	12.3 ± 6.8	18.7 ± 7.2	-34.2	< 0.001
	**Orthostatic hypotension (n, %)**	35 (58.3%)	2 (6.7%)	+94.3	<0.001

Abbreviations: BRS, Baroreflex Sensitivity; DBP, Diastolic Blood Pressure; E:I, Expiration to Inspiration ratio; HR, Heart Rate; RMSSD, Root Mean Square of Successive Differences; SBP, Systolic Blood Pressure; SDNN, Standard Deviation of NN intervals.

Notes: Data presented as mean ± SD or count (percentage). Between-group comparisons performed using independent t-tests for continuous variables and Chi-squared test for categorical variables. All autonomic parameters were measured in the medication OFF state for PD patients.

Adrenergic function assessment through orthostatic challenge revealed significant sympathetic vasoconstrictor impairment. During tilt testing, PD patients experienced more than double the systolic blood pressure drop versus controls, with diastolic blood pressure similarly affected. The prevalence of orthostatic hypotension was 58.3% in PD patients versus 6.7% controls. Compensatory heart rate increase during orthostatic stress was blunted by 34.2% in PD patients, suggesting inadequate chronotropic compensation.

Correlation analyses between demographic and clinical variables revealed that disease duration significantly correlated with H&Y stage (r = 0.58; *p* < 0.001) and UPDRS III score (r = 0.45; *p* < 0.001). Age showed weak correlations with SDNN (r = -0.28; *p* = 0.031) and baroreflex sensitivity (BRS) (r = -0.25; *p* = 0.048). Body mass index did not significantly correlate with any autonomic parameters (all *p* > 0.05). Sex differences in autonomic parameters were not statistically significant after adjusting for age and disease severity (all *p* > 0.05).

Significant associations emerged between autonomic parameters and clinical severity indicators ( Table 3 ). Heart rate variability demonstrated inverse relationships with motor severity and disease stage, remaining robust after controlling for age, disease duration, and medications. Baroreflex sensitivity showed progressive deterioration with advancing stage and motor burden, maintaining significance in multivariate analyses.

**Table 3:** Correlations between autonomic parameters and disease severity markers in PD patients

Autonomic parameter	Disease severity marker	r	*p* -value	Partial r*	*p* -value*
**SDNN**	UPDRS III score	-0.52	< 0.001	-0.47	< 0.001
**SDNN**	H&Y stage	-0.41	0.001	-0.38	0.003
**BRS**	H&Y stage	-0.48	< 0.001	-0.43	0.001
**BRS**	UPDRS III score	-0.44	< 0.001	-0.40	0.002
**SBP drop on tilt**	Disease duration	0.43	< 0.001	0.38	0.003
**SBP drop on tilt**	LEDD	0.37	0.004	0.32	0.013
**Valsalva ratio**	Disease duration	-0.36	0.005	-0.31	0.017
**HR response to deep breathing**	UPDRS III score	-0.39	0.002	-0.34	0.008

Abbreviations: BRS, baroreflex sensitivity; HR, heart rate; H&Y, Hoehn and Yahr; LEDD, levodopa equivalent daily dose; SBP, systolic blood pressure; SDNN, standard deviation of NN intervals; UPDRS, Unified Parkinson's Disease Rating Scale.

Notes: Pearson's correlation coefficients (r) and partial correlation coefficients (partial r) are shown. Partial correlations were adjusted for age, disease duration (when disease duration was not the variable being correlated), and LEDD (when LEDD was not the variable being correlated). All correlations performed using autonomic parameters measured in the medication OFF state.

Orthostatic blood pressure dysregulation positively correlated with disease duration, suggesting worsening sympathetic dysfunction over time. The magnitude of orthostatic drop also correlated with higher levodopa equivalent daily dose, potentially reflecting more advanced disease or medication contribution to orthostatic hypotension. Valsalva ratio declined progressively with longer disease duration, while heart rate response to deep breathing correlated inversely with motor severity. These correlations support autonomic dysfunction as integral to PD pathophysiology, worsening with progression.

Comparison between medication OFF and ON states revealed modest but significant improvements ( Table 4 ). Heart rate variability measures showed significant ON-state improvements: SDNN increased 14.6% and RMSSD 14.5%, suggesting enhanced parasympathetic modulation. Baroreflex sensitivity demonstrated similar 15.4% improvement. However, heart rate response to deep breathing showed non-significant trend, and Valsalva ratio showed minimal change.

**Table 4:** Effect of dopaminergic medication on autonomic parameters in PD patients

Parameter		OFF state	ON state	Change (%)	*p* -value
**Cardiovagal function**	SDNN (ms)	19.8 ± 7.9	22.7 ± 8.5	+14.6	0.032
	RMSSD (ms)	17.3 ± 6.8	19.8 ± 7.4	+14.5	0.045
	BRS (ms/mmHg)	3.9 ± 1.6	4.5 ± 1.9	+15.4	0.041
	HR response to deep breathing (bpm)	7.8 ± 3.0	8.7 ± 3.3	+11.5	0.058
	Valsalva ratio	1.19 ± 0.14	1.23 ± 0.15	+3.4	0.087
**Adrenergic function**	SBP drop on tilt (mmHg)	24.3 ± 13.2	20.8 ± 12.3	-14.4	0.025
	DBP drop on tilt (mmHg)	12.7 ± 7.8	11.1 ± 7.2	-12.6	0.142
	HR increase on tilt (bpm)	11.8 ± 6.5	12.9 ± 6.9	+9.3	0.216
	Orthostatic hypotension (n, %)	37 (61.7%)	32 (53.3%)	+13.5	0.321

Abbreviations: BRS, baroreflex sensitivity; DBP, diastolic blood pressure; HR, heart rate; RMSSD, root mean square of successive differences; SBP, systolic blood pressure; SDNN, standard deviation of NN intervals.

Notes: Data presented as mean ± SD or count (percentage). Within-subject comparisons were performed using paired t-tests. Percentage change calculated as: [(ON state - OFF state) / OFF state] × 100.

Orthostatic blood pressure regulation modestly improved in ON state, with systolic blood pressure drop during tilt testing reduced by 14.4%. Despite improvement, orthostatic hypotension prevalence remained high: 61.7% in OFF state versus 53.3% in ON state. Diastolic blood pressure drop and heart rate response to tilt showed non-significant improvement trends. These findings suggest that dopaminergic medication provides some benefit but does not normalize function or eliminate orthostatic hypotension in most patients.

Exploratory analyses stratified by medication type suggested combination therapy (levodopa plus dopamine agonist, n = 28) produced more pronounced cardiovagal improvements versus levodopa monotherapy (n = 23), with SDNN increasing 18.3% versus 9.7% respectively. However, small subgroup sizes limit interpretation, requiring confirmation in larger studies.

## DISCUSSION

The current study provides comprehensive evidence of widespread cardiovascular autonomic dysfunction in PD, with significant impairment across parasympathetic and sympathetic domains. Strong correlations between autonomic dysfunction and disease severity, plus modest dopaminergic therapy effects, have important implications for understanding pathophysiology and management. The profound dysfunction observed with 34 to 41% reductions in cardiovagal parameters and nearly threefold increased orthostatic blood pressure drop underscores pervasive nature of autonomic involvement. 16 17 The 34.5% heart rate variability reduction reflects impaired autonomic cardiac modulation in PD. 35 36 This is clinically significant as low heart rate variability is associated with increased cardiovascular morbidity and mortality. 37

The BRS impairment, with 40.8% reduction, represents a critical finding. Baroreflex provides rapid blood pressure stabilization through compensatory heart rate adjustments. 18 Impaired baroreflex function compromises cardiovascular homeostasis, contributing to blood pressure instability including orthostatic hypotension and supine hypertension. 38 High orthostatic hypotension prevalence (58.3%) aligns with epidemiological reports of 30 to 60% in PD populations. 39 The 171% greater orthostatic blood pressure drop compared to controls reflect significant sympathetic vasoconstrictor impairment, resulting from cardiac sympathetic denervation and impaired peripheral noradrenergic neurotransmission extensively documented through imaging and biochemical studies. 19

Significant correlations between autonomic parameters and disease severity provide compelling evidence that autonomic dysfunction is integral to PD pathology, progressing parallel to motor symptoms. The strong negative correlation between heart rate variability and UPDRS III scores (r = -0.52) suggests shared pathophysiological mechanisms, potentially reflecting widespread alpha-synuclein pathology affecting nigrostriatal dopaminergic pathways and autonomic regulatory centers. 6

Baroreflex sensitivity correlation with H&Y stage indicates progressive autonomic impairment through clinical stages, supporting the Braak hypothesis of predictable alpha-synuclein spread affecting autonomic structures early and progressively involving extensive networks. 7 Positive correlation between orthostatic hypotension severity and disease duration emphasizes progressive autonomic deterioration, potentially reflecting ongoing pathological deposition, neuroinflammation, and cumulative neuronal loss. 11

Modest but significant improvements in autonomic parameters during ON state (14–15% in heart rate variability and baroreflex sensitivity) provide insights into complex dopaminergic-autonomic interactions. Observed cardiovagal improvements may reflect multiple mechanisms. Dopamine directly modulates autonomic control centers including nucleus tractus solitarius and dorsal motor nucleus of vagus, critical for cardiovascular regulation. Medication may partially enhance these centers' function. Improvements may also be indirectly mediated through enhanced motor function reducing metabolic demands and sympathetic activation, allowing improved parasympathetic tone. 12

Although the pattern of ON-state changes was not uniform across autonomic domains, suggesting a pathway-specific effect of dopaminergic therapy, improvements were more consistently observed in cardiovagal indices, such as heart rate variability and baroreflex sensitivity. Whereas the parameters predominantly reflecting sympathetic adrenergic vasomotor function, including orthostatic blood pressure control, remained substantially impaired. This dissociation supports the concept that dopaminergic therapy preferentially modulates central autonomic circuits involved in cardiovagal control, while peripheral sympathetic noradrenergic dysfunction, known to underlie orthostatic hypotension in PD, appears less responsive to dopaminergic replacement.

Variable effects across parameters suggest complex, pathway-specific dopaminergic influences. While cardiovagal parameters showed significant improvements, adrenergic function effects (orthostatic blood pressure control) were modest, potentially reflecting predominant noradrenergic rather than dopaminergic mechanisms in blood pressure regulation. Importantly, despite significant improvements, autonomic parameters remained substantially impaired versus controls, indicating dopaminergic medication provides only partial compensation and highlighting need for additional therapeutic strategies targeting autonomic symptoms. 14

### Clinical implications

#### *Routine autonomic assessment*

Given high prevalence and clinical impact, systematic autonomic assessment should be routine in PD patients. Simple bedside tests such as orthostatic vital signs should be performed regularly, with comprehensive testing for symptomatic autonomic dysfunction or unexplained symptoms like falls, syncope, or fatigue.

#### *Disease severity marker*

Strong correlations suggest autonomic assessment provides valuable disease monitoring and prognostication information for disease severity. Autonomic markers might complement traditional motor assessments in trials and serve as therapeutic intervention endpoints.

#### *Therapeutic considerations*

Modest dopaminergic medication benefits suggest optimization may extend beyond motor control. However, persistent impairment highlights need for specific autonomic symptom treatments. Non-pharmacological interventions (dietary modifications, compression garments, physical maneuvers) and pharmacological therapies (midodrine, droxidopa, fludrocortisone) should be considered for symptomatic orthostatic hypotension.

#### *Medication management*

Clinicians must balance dopaminergic therapy benefits against potential orthostatic hypotension exacerbation. Some medications, particularly dopamine agonists, may worsen orthostatic hypotension through peripheral vasodilation. Our finding of modest ON-state improvement suggests benefits generally outweigh risks, but individual responses require careful monitoring.

The comprehensive autonomic dysfunction observed in this study, involving both cardiovagal and adrenergic systems without selective vulnerability, suggests widespread alpha-synuclein pathology affecting multiple autonomic nervous system hierarchy levels. Recent research demonstrates extensive cardiac sympathetic denervation in PD, detectable through meta-iodobenzylguanidine scintigraphy, likely contributing to reduced heart rate variability and impaired orthostatic responses observed in our study. Peripheral autonomic pathology extends beyond cardiac innervation to sympathetic ganglia, enteric nervous system, and other peripheral structures.

Correlation between autonomic dysfunction and disease severity may reflect common underlying pathological processes driven by alpha-synuclein aggregation. According to Braak staging, pathology may begin peripherally in autonomic structures and olfactory system before spreading to substantia nigra, suggesting autonomic dysfunction might not only parallel but potentially precede motor symptoms, serving as early biomarker.

### Limitations and future directions

Cross-sectional design limits causal inference and longitudinal progression tracking. Key autonomic domains (gastrointestinal, sudomotor) were not comprehensively evaluated, possibly underestimating overall involvement. Single-center recruitment restricts generalizability to broader populations. Future research should prioritize prospective longitudinal studies clarifying temporal relationships between autonomic impairment and disease progression, evaluating whether autonomic markers predict trajectory or treatment responses. Studies focusing on prodromal PD symptoms (rapid eye movement [REM] sleep behavior disorder, hyposmia) could determine if autonomic dysfunction precedes motor symptoms, potentially serving as early diagnostic biomarker. Expanding testing to include sudomotor, pupillomotor, and visceral assessments would provide comprehensive autonomic impairment evaluation.

In conclusion, the current study demonstrates widespread cardiovascular autonomic dysfunction in PD, with substantial parasympathetic and sympathetic impairments correlating strongly with disease severity. Dopaminergic medication showed modest but significant benefits; however, dysfunction persisted in the ON state, indicating need for additional targeted therapies. Findings underline routine autonomic assessment importance in PD and suggest autonomic measures as valuable disease progression markers. Further longitudinal studies are warranted exploring underlying mechanisms, developing early diagnostic biomarkers, and evaluating specific interventions to improve patient outcomes.
